# Research on risk decision-making generation method for water conservancy project based on multimodal knowledge graph and large language model

**DOI:** 10.1371/journal.pone.0330258

**Published:** 2025-08-26

**Authors:** Libo Yang, Yuan Li, Junhua Tan, Libo Mao

**Affiliations:** 1 Advanced Research Institute for Digital-Twin Water Conservancy, North China University of Water Resources and Electric Power, Henan, Zhengzhou, China; 2 School of Information Engineering, North China University of Water Resources and Electric Power, Henan, Zhengzhou, China; Hunan University, CHINA

## Abstract

Traditional knowledge graphs of water conservancy project risks have supported risk decision-making. However, they are constrained by limited data modalities and low accuracy in information extraction. A multimodal water conservancy project risk knowledge graph is proposed in this study, along with a synergistic strategy involving multimodal large language models Risk decision-making generation is facilitated through a multi-agent agentic retrieval-augmented generation framework. To enhance visual recognition, a DenseNet-based image classification model is improved by incorporating single-head self-attention and coordinate attention mechanisms. For textual data, risk entities such as locations, components, and events are extracted using a BERT-BiLSTM-CRF architecture. These extracted entities serve as the foundation for constructing the multimodal knowledge graph. To support generation, a multi-agent agentic retrieval-augmented generation mechanism is introduced. This mechanism enhances the reliability and interpretability of risk decision-making outputs. In experiments, the enhanced DenseNet model outperforms the original baseline in both precision and recall for image recognition tasks. In risk decision-making tasks, the proposed approach—combining a multimodal knowledge graph with a multi-agent agentic retrieval-augmented generation method—achieves strong performance on BERTScore and ROUGE-L metrics. This work presents a novel perspective for leveraging multimodal knowledge graphs in water conservancy project risk management.

## 1. Introduction

China’s water resources are highly uneven in spatial and temporal distribution, with water shortages particularly severe in northern regions. The South-to-North Water Diversion Project has played a vital role in alleviating water scarcity, ensuring urban and rural water supply security, and promoting regional coordinated development [1]. With the continuous advancement of water conservancy informatization, artificial intelligence(AI) technologies have been widely applied to the operation and management of water conservancy projects, significantly improving efficiency and safety. However, long-term operation has led to increasing issues such as infrastructure aging and equipment deterioration. These problems have intensified operational risks and imposed higher demands on AI for risk identification and decision-making support. In particular, the ability to deliver rapid, accurate, and comprehensive responses during risk events has become a critical requirement for ensuring the safe operation of the water conservancy infrastructure.

In intelligent risk management systems, risk decision-making generation enables the provision of scientific response strategies at the earliest stage of an emergency. Compared to traditional risk handling methods that rely on manual expertise, AI-driven decision-making systems offer advantages in terms of systematization, comprehensiveness, and scalability. As a key tool for modeling risk information, knowledge graphs have played a fundamental role in the early stages of water conservancy risk management. [2] developed a knowledge graph for emergency response planning in water conservancy projects that supports the automated retrieval of response plans under risk scenarios. [3] proposed a flood control and emergency response knowledge graph, which provided reliable knowledge support for emergency analysis.

With the increasing diversity and heterogeneity of risk data sources, the construction of multimodal knowledge graphs has become a key research focus [4]. [5] built a multimodal knowledge graph for water diversion projects by integrating image and textual information. [6] combined remote sensing imagery with social media text for flood disaster knowledge modeling. However, in the construction of such graphs, image data are often overlooked. Moreover, water conservancy imagery exhibits significant variations across different risk regions. Traditional convolutional neural networks (CNNs) struggle to maintain consistency between global and local semantics under these conditions. In addition, the presence of redundant background information severely hinders the accurate extraction of effective risk features. Therefore, improving the adaptability and accuracy of image recognition models is a critical prerequisite for building high-quality multimodal knowledge graphs in the water conservancy domain.

In this study, the primary types of risks addressed include structural risks (e.g., cracks, leakage, settlement), natural disaster-related risks (e.g., rainfall-induced gully, sedimentation), and management-related risks (e.g., equipment aging, gate failures, and improper operation). These risks are commonly observed in the inspection records of the Middle Route Project of the South-to-North Water Diversion and form the basis for constructing the multimodal water conservancy project risk knowledge graph.

For complex, multi-source risk reasoning tasks, the emergence of Multimodal Large Language Models (MLLM) [7] has opened new avenues for knowledge acquisition and decision-making generation. Models such as VideoLLaMA3 [8], ViP-LLaVA [9], and Qwen-VL2.5 [10] demonstrate strong multimodal reasoning capabilities by integrating text, image, and other modalities [11] applied LLM in conjunction with prompt chaining [12] to conduct flood emergency reasoning, showcasing the potential of next-generation knowledge generation frameworks. However, current LLM still face the challenge of hallucination, where generated content lacks domain constraints [13]. Although incorporating external vector databases can partially mitigate this issue, these databases often lack semantic association and hierarchical modeling capabilities, limiting their effectiveness. To address this, this study proposes the use of a multimodal water conservancy risk knowledge graph as an external knowledge source for MLLM. An AI agent mechanism [14] is further introduced to construct a Multi-Agent Agentic Retrieval-Augmented Generation (MAAR) strategy [15], aiming to enhance the completeness and scientific reliability of risk responses. Based on the above analysis, the core objectives and contributions of this study are summarized as follows: (1) To improve the risk image recognition model and enhance recognition accuracy in complex backgrounds; (2) To construct a multimodal water conservancy project risk knowledge graph that integrates multi-source heterogeneous information; (3) To propose a MAAR strategy that fuses the multimodal knowledge graph with MLLM, thereby optimizing intelligent decision-making and generation quality for risk response.

## 2. Multimodal water conservancy project risk knowledge extraction

### 2.1. DenseNet-based risk image recognition for water resources projects

Due to differences in the material properties of hydraulic structures and natural objects, water reflections, vegetation, and cloud cover introduce significant background redundancy. Risk-related targets such as cracks, sediment accumulation, and equipment damage are often embedded in visually complex backgrounds. These factors interfere with the feature extraction precision of traditional CNNs, making it difficult to locate risk regions under cluttered scenes. Although Transformer-based networks with self-attention mechanisms [16] have been applied, they struggle to simultaneously capture global structures and local risk details in water conservancy imagery. Furthermore, directly applying Transformers to high-resolution water-related imagery often results in excessive computational overhead. Therefore, for complex water conservancy risk scenarios, a hybrid architecture combining CNNs with self-attention mechanisms can more effectively capture and identify risk information.

#### 2.1.1. DenseNet.

DenseNet [17] consists of multiple dense blocks and transition layers. Each block adopts a fully connected pattern, where the output of every preceding layer is passed to all subsequent layers. For layer n , its feature representation $x_{1}$ is defined by the following core formulation:


$$x_{1}=H_{n}\left(\left[x_{0},x_{1},\ldots ,x_{n-1}\right]\right)$$ (1)

In this formulation, $H_{n}$ denotes the transformation function at layer n, which includes Batch Normalization, ReLU activation, and convolution operations. $\left[x_{0},x_{1},\ldots ,x_{n-1}\right]$ represents the concatenation of feature maps from all preceding layers.

Due to the concatenation structure within each dense block, the number of feature channels increases rapidly as the network deepens, leading to higher computational complexity. To mitigate this, transition layers are inserted between blocks to perform dimensionality reduction. Assuming the input channel number is Cin, the output channel number after a transition layer is computed as:


Cout=[θCin] (2)

In the formula: θ is the scaling down ratio.

**Fig 1:**
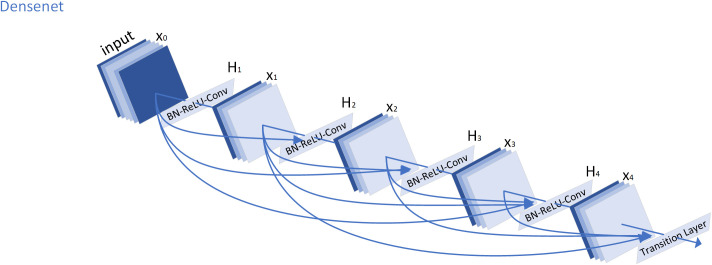
Densenet Architecture.

In this study, a variant of DenseNet, namely DenseNet-121, is adopted and further improved. The network comprises 121 layers, with the dense block configuration set as (6, 12, 24, 16). Each dense block contains multiple units, and each unit consists of two sequential operations: Batch Normalization, ReLU activation, and convolution. The transition layers, which connect adjacent blocks, comprise Batch Normalization, ReLU, convolution, and an average pooling operation that reduces the number of channels by half. As shown in Fig 1, the overall architecture of the DenseNet network is illustrated.

#### 2.1.1. Improved DenseNet.

To address the specific characteristics of water conservancy risk imagery, a Single-Head Self-Attention (SHSA) mechanism [18] is introduced to suppress the influence of irrelevant regions. This mechanism enhances the network’s focus on key risk areas such as dam cracks, gate failures, and abnormal water flow. Within the original DenseNet architecture, integrating SHSA into the layered feature extraction process enables unified semantic representation across modalities. This integration improves feature extraction accuracy and the network’s understanding of complex hydrological scenarios. In the SHSA layer, the input feature channels are divided into two parts. One part undergoes spatial feature aggregation via the single-head attention mechanism, while the other remains unchanged. The computation process of the SHSA layer is defined as follows:


$$\left\{ \begin{array}{c} SHSA(X)=Concat({\tilde{X}}_{attb}X_{res})W^{0}\\ {\tilde{X}}_{att}=Attention(X_{att}W^{Q},X_{att}W^{K},X_{att}W^{V})\\ Attention(Q,K,V)=Softmax\left(QK^{T}/\sqrt{d_{qk}}\right)V\\ X_{att},X_{res}=Split(X,[C_{p},C-C_{p}]) \end{array}\right.$$ (3)

In the equation, $W^{Q}$ $W^{K}$$W^{V}$ and $W^{0}$ are the projection weights, which $d_{qk}$ are the dimensions of the query and keyword, Concat is the connection operation and $C_{p}$ is the initial channel.

For key risk regions, a Coordinate Attention (CoordAtt) mechanism [19] is incorporated. This mechanism encodes spatial coordinate information and integrates it into the channel attention process. It enables the network to focus more precisely on target regions while suppressing background interference. Specifically, feature maps generated by previous modules are first concatenated and passed through a shared 1 × 1 convolution to produce an intermediate feature map $f\in R^{C/r\times (H+W)}$, where r is the downsampling ratio that controls the size of the module. Next, the feature map f is split along the spatial dimension into $f^{h}\in R^{C/r\times H}$ and $f^{w}\in R^{C/r\times W}$, which are each transformed back to the original number of channels using 1 × 1 convolutions. Finally, the outputs are expanded and used to generate attention weights $g^{h}$ and $g^{w}$. The corresponding equations are as follows:


$$\left\{ \begin{array}{c} f=\delta \left(F\left(\left[Z^{h},Z^{w}\right]\right)\right)\\ g^{h}=\sigma \left(F_{h}(f^{h})\right)\\ g^{w}=\sigma \left(F_{w}(f^{w})\right)\\ y_{c}(i,j)=x_{c}(i,j)\times g_{c}^{h}(i)\times g_{c}^{w}(j) \end{array}\right.$$ (4)

In the equations, $Z^{h}$ is the channel with height h, $Z^{w}$ is the channel with height w, $F_{h}$ and $F_{w}$ are convolution operations.

**Fig 2:**
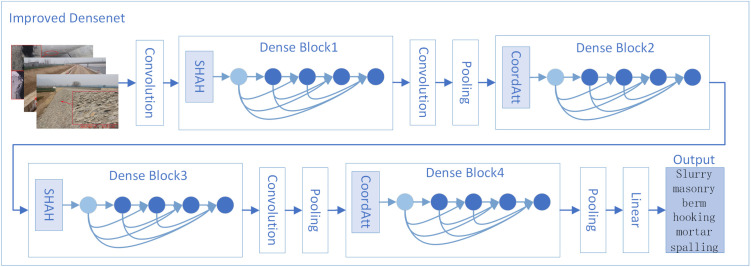
Improved DenseNet Architecture.

Finally, the SHSA and CoordAtt modules are embedded into the stage-wise backbone of the convolutional architecture of DenseNet-121. Based on the convolutional stage configuration (6, 12, 24, 16), each module is positioned to maximize its functional contribution. To address the issue of class imbalance in training samples, Focal Loss [20] is adopted as the objective function. This approach emphasizes hard negative samples to improve training stability and balance model performance. The improved network architecture consists of three components: a feature extraction layer, an SHSA-CoordAtt alternating fusion layer, and a risk image recognition layer for water conservancy projects. Fig 2 illustrates the improved DenseNet-SHSA-CoordAtt model, which incorporates an alternating fusion strategy based on SHSA and CoordAtt mechanisms.

### 2.2. Entity recognition model based on BERT+BiLSTM+CRF

Risk entities in water conservancy projects are often embedded in large volumes of inspection text. Due to differences in inspectors’ professional backgrounds and education levels, the same risk event may be described using diverse expressions. Multiple risk terms often refer to a single standardized concept, which increases the difficulty of accurate entity extraction. Therefore, precisely identifying risk time, location, affected components, and risk events within the text is critical. However, knowledge expression patterns must be learned from limited inspection data. This results in insufficient semantic representation capabilities and a lack of specialized hydrological dictionaries,

which hinder effective entity extraction [2]. To address these issues, this study employs a BERT+BiLSTM+CRF model to identify entities in inspection texts.

This paper adopts the BIO tagging scheme [21] to annotate entities in the text. “B” indicates the beginning of an entity, “I” indicates that the character is inside an entity, and “O” means that the character does not belong to any entity. Among them, T, L, P, and E represent risk time, risk location, risk part, and risk event, respectively. For example, in the sentence: “On March 13, during an inspection, it was found that the bottom of the sluice gate on the left bank of the Penghe Aqueduct in Section 2 of Lushan South was leaking.” “March” and”13” are labeled as risk time. “Lushan South,” “Section 2”, “Penghe,” and “left bank of the aqueduct” are uniformly labeled as risk locations. “Sluice gate” and “gate” are understood as the risk part, and “leaking” represent the risk event. Table 1 details the risk inspection text sequence, label sequence, and corresponding entity information.

**Table 1:** Example of Labeling.

Text	On	March	13	,	during	an	inspection	,	it	was	found	
Label	O	B-T	I-T	O	O	O	O	O	O	O	O	
Entity	RiskTime,L											
Text	that	the	bottom	of	the	sluice	gate	on	the	left	bank	of
Label	O	O	B-P	I-P	I-P	I-P	I-P	O	O	B-L	I-L	I-L
Entity			RiskPart,P							RiskLocation,L		
Text	the	Penghe	Aqueduct	in	Section	2	of	Lushan	South	was	leaking	.
Label	I-L	I-L	I-L	O	B-L	I-L	I-L	I-L	I-L	O	B-E	O
Entity	RiskLocation,L									RiskEvent,E		

**Fig 3:**
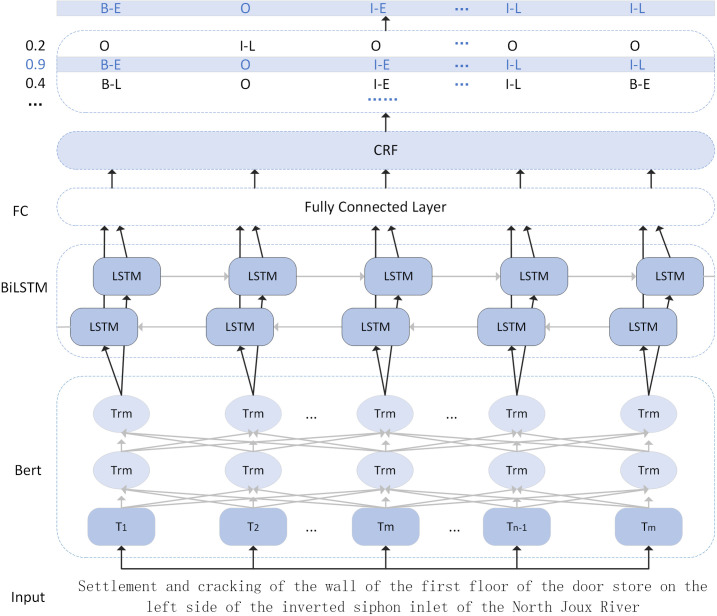
BERT+BiLSTM+CRF architecture.

First, the pre-trained BERT model [22], originally trained on large-scale general-purpose corpora, is transferred to the domain of water conservancy engineering. The model is then fine-tuned using domain-specific data to improve the accuracy of entity extraction in complex hydrological scenarios. For example, given the input sentence “Settlement and cracking occurred on the first-floor wall of the left-side gate chamber at the Beiru River inverted siphon inlet,” the model follows a multi-stage processing pipeline. The sentence is first encoded by the BERT encoder layer, which converts the textual input into high-dimensional semantic vectors interpretable by the model. Next, these vectors are passed to a BiLSTM network, which captures bidirectional dependencies within the risk-related text sequence. This step enhances the model’s contextual understanding. The resulting representations are fed into a fully connected (FC) layer, which maps the character-level embeddings to a predefined label space of hydrological risk entities. Finally, a Conditional Random Field (CRF) [23] layer is applied to optimize the sequence of predicted labels, ensuring the output sequence is globally coherent and accurate. The detailed architecture of the BERT+BiLSTM+CRF model is illustrated in Fig 3.

## 3. Multimodal water conservancy project risk knowledge graph construction

**Fig 4:**
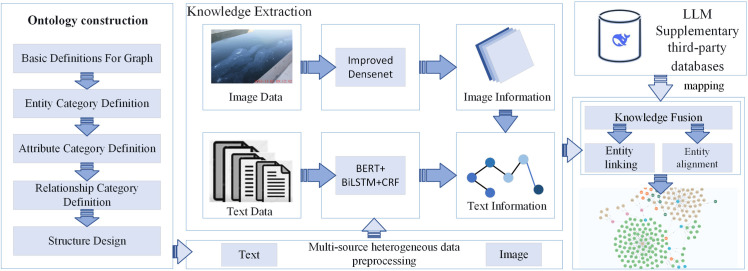
Multimodal Water Conservancy Project Risk Knowledge Graph Construction Process.

The Middle Route Project of the South-to-North Water Diversion is a key component of China’s national water transfer initiative. It aims to alleviate severe water scarcity across the Huang-Huai-Hai Plain [24]. This project involves numerous structures and extended pipelines, encompassing diverse engineering forms. During operation, it is subject to multiple risks, including structural risks, natural disasters, and management-related issues. Based on recent inspection risk data from the Middle Route Project, this study constructs a multimodal knowledge graph for water conservancy project risks. To reduce the impact of risk events, the construction of the multimodal knowledge graph follows five main phases: ontology design, multi-source heterogeneous data preprocessing, knowledge extraction, knowledge fusion, and graph storage. The detailed workflow is illustrated in Fig 4.

### 3.1. Ontology construction

From the perspective of risk decision-making generation, risk-related imagery provides substantial supplementary information during the generation process. Water conservancy project risk events contain a wealth of implicit information, including the time of risk discovery, the reporting personnel, risk severity level, and detailed image-text descriptions. Risk contingency plans, guided by the Risk Prevention and Control Manual, outline potential consequences and corresponding mitigation measures for each risk type. The identification of risk sources, consequence definitions, and standard references for related engineering projects are based on official guidelines, including operational risk identification protocols, major production safety hazard regulations, and risk assessment and management standards.

**Fig 5:**
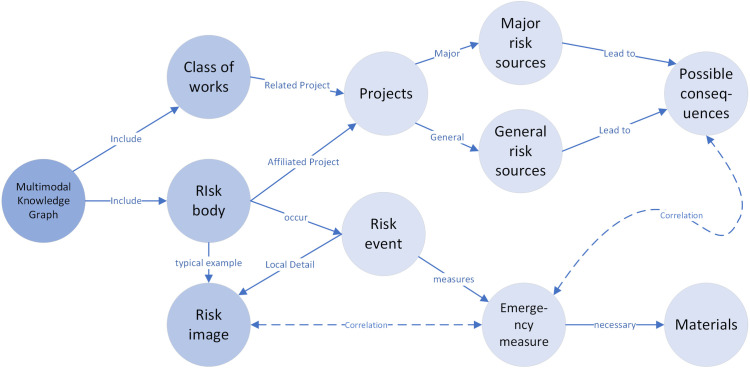
Ontology Modeling of Multimodal Water Conservancy Project Risk Knowledge Graph.

The risk inspection records from the Middle Route Project are real-world cases documented by field personnel. Based on this information, the multimodal water conservancy project risk knowledge graph ontology is constructed, focusing on three core conceptual relationships: (1) engineering-related components, (2) events and response measures linked to risk subjects, and (3) representative risk images associated with specific structural components. This study explores risk events through three key elements—subject, component, and event—and classifies various risk scenarios accordingly. The identified event categories are abstracted and used as critical retrieval cues during the risk decision-making generation process. The ontology modeling results are shown in Fig 5.

Each type of engineering project (such as pumping stations, power plants, embankments, etc.) is associated with corresponding risk body. Risk body refer primarily to the objects where specific risks occur or are likely to occur. Based on past expert experience, the sources of risk within these projects are categorized into two types: major risk sources and general risk sources. The possible consequences of different levels of risk sources are closely related to the corresponding emergency response measures. Identical risks are generally associated with the same emergency measures. When a specific risk body experiences a relevant risk event, appropriate emergency measures are adopted accordingly. Materials refer to the general term for items required during the implementation of emergency measures, including but not limited to equipment, tools, consumables, and resources. Risk images are mainly used to illustrate examples of risk body. For those risk body where risk events have occurred, localized details are intuitively presented through risk images. The risk descriptions in the images share similarities with actual risk events, and by linking emergency measures to them, a complete relationship is established.

### 3.2. Multi-source heterogeneous data preprocessing

Different risk events may occur for the same risk subject. Therefore, before processing the risk imagery, reclassification based on expert knowledge in the water conservancy domain is required. Due to differences in reporters and imaging devices, the original sizes of water conservancy project risk images vary significantly [5]. To improve processing efficiency and accelerate model training, all images are uniformly resized to 224 × 224 pixels. Image augmentation techniques such as flipping, mirroring, and sharpening are applied to expand the dataset. The inspection records of the South-to-North Water Diversion Project are primarily completed manually. As a result, human errors such as spelling mistakes, grammatical issues, and duplicate entries are inevitable. The main task in text preprocessing is noise reduction, which aims to eliminate irrelevant information and enhance the overall quality of textual data. Table 2 shows some examples of inspection records.

**Table 2:** Examples of inspection records.

Location	Qualitative Risk Level	Risk Description
Section 1 – Cixian South Main Canal	Moderate to High	One transverse crack detected in the lining panel immediately downstream of the inlet structure at the off-take.
Section 3 – Xinwei Xiangquan River	Moderate to High	Water leakage observed at the right-side seal of Gate #1 (radial gate) of the canal inverted siphon.
148 + 002–148 + 006	Low to Moderate	freeze–thaw scaling on the concrete lining panel, measuring approx. 4 m (L) × 0.5 m (W).

### 3.3. Entity relationship extraction

The improved DenseNet is employed to extract risk features from images. The results are then converted into textual representations of risk event entities. For textual data, the BERT+BiLSTM+CRF model is applied to extract entities such as risk location, affected component, and risk event from inspection records. A dictionary-driven relation extraction method is adopted due to its strong adaptability. By constructing a verb lexicon and syntactic patterns, the method can directly identify relationships between entities from the text [25]. In the context of water conservancy project risks, entity relationships are often based on simple logical structures. Therefore, this study defines and detects these relationships using explicit semantic patterns and rule-based strategies. In this study, relation extraction is performed using a pattern matching-based entity co-occurrence analysis method. Taking the sentence “The expansion joint between Span 2 and Span 3 of the Left Discharge Aqueduct in Front of Jiangzhai, Section 1 of Xingyang is leaking” as an example, the process begins with explicit relation matching, wherein the presence of both a risk entity and a risk event is identified. If both are detected within the same sentence, a relation triple is directly generated. For instance: (“Span 2 and Span 3 of the Left Discharge Aqueduct”, “occurs”, “expansion joint leakage”). Following this, implicit relation matching is conducted based on the sequential order of entities. The first extracted entity is treated as the head entity, and each subsequent entity forms a candidate entity pair with it, serving as the tail entity. If a candidate pair such as (“Front of Jiangzhai, Section 1 of Xingyang”, “Span 2 and Span 3 of the Left Discharge Aqueduct”) matches a predefined standard co-occurrence pattern (e.g., “Location”, “Risk Entity”), the corresponding relation “exists” is assigned. Consequently, a new relation triple is generated: (“Front of Jiangzhai, Section 1 of Xingyang”, “exists”, “Span 2 and Span 3 of the Left Discharge Aqueduct”).

### 3.4. Knowledge fusion

Entity linking refers to the process of connecting entities from different data sources. Entity alignment involves mapping entities that describe the same object across heterogeneous datasets [26]. Both tasks are essential for improving the quality of the multimodal water conservancy project risk knowledge graph. After extracting risk entity relations, the goal is to obtain structured data in the form of “entity–relation–entity” and “entity–attribute–value” triples. Integrating text-based entities derived from risk imagery with those extracted from inspection texts is a key challenge. To address this, BERT is used to compute the semantic similarity between two entity labels [27]. A predefined threshold is then applied to determine whether a matched label pair should be linked to the knowledge graph. Specifically, the risk image label T1 and the text label T2 are input into the BERT model. The [CLS] token outputs $h_{1}$ and $h_{2}$ are used as the semantic representations of each label.


$$h_{i}=[h_{i}0,h_{i}1,\ldots ,h_{i}d]$$ (5)

In the equation, d is the dimension of the hidden layer of the model.

Cosine similarity is then used to compute the semantic similarity between the two label representations.


$$Sim(T_{1},T_{2})=\frac{h_{1}\cdot h_{2}}{∥h_{1}∥\cdot ∥h_{2}∥}$$ (6)

In the equation, $h_{1}\cdot h_{2}$ denotes the vector dot product and $∥h_{1}∥$ $∥h_{2}∥$ denote the L2 paradigm of the vector, respectively.

### 3.5. Graph storage

Neo4j is a native graph database designed for storing and querying graph-structured data. It enables intuitive visualization of relationships among entities. The structured triples representing water conservancy project risks contain rich information. This study adopts the Neo4j graph database to store and manage these data efficiently. The extracted triples are imported using the Py2neo library, resulting in a multimodal water conservancy project risk knowledge graph containing 14,228 entities. Each entity node is assigned a unique identifier during the storage process. To further enhance the quality of the knowledge graph, MLLM is employed to screen out potentially irrelevant nodes during triple construction. The finalized graph is then subjected to a verification process.

## 4. Application of multimodal water conservancy project risk knowledge graph

LLM is trained on extensive public datasets but lacks domain-specific or proprietary information access. This limitation may prevent LLM from providing comprehensive or accurate responses in specialized domains. Retrieval-augmented Generation (RAG) [28] addresses this issue by retrieving relevant information from an external knowledge base during the generation process. By doing so, RAG enables LLM to generate informed answers even when internal training data are insufficient. This mitigates the risk of generating inaccurate or hallucinated content. Moreover, RAG enhances the interpretability of the generated results by grounding them in explicit external sources. To fully leverage the multimodal water conservancy project risk knowledge graph, this study integrates a state-of-the-art MLLM with the RAG framework. The proposed solution, or MAAR, is designed to support the generation of risk contingency plans for water conservancy projects. This implementation uses the locally deployed Qwen2.5-VL-7B model as the decision engine. This model can identify risks depicted in water conservancy imagery and perform reasoning over input text to support informed decision-making.

### 4.1. Risk decision-making generation by fusing multimodal water conservancy project risk knowledge graph and MLLM

**Fig 6:**
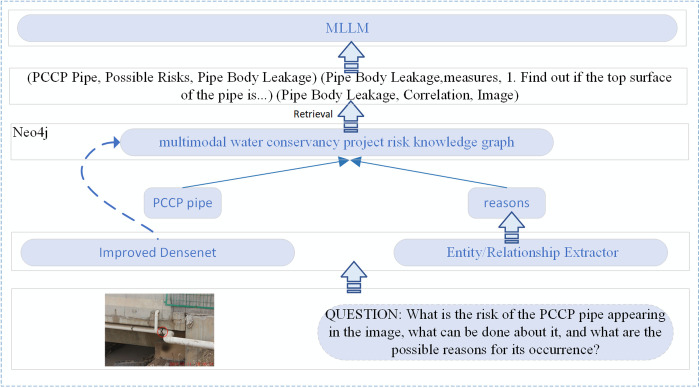
Enhanced MLLM for Multimodal Water Conservancy Project Risk Knowledge Graph.

During the operation and management of the Middle Route Project, a large volume of multimodal risk data has been accumulated. The multimodal water conservancy project risk knowledge graph constructed from these data enables external knowledge retrieval, which can guide and constrain the generation process of MLLM. This approach significantly improves the accuracy of model responses within the domain of water conservancy risk. Integrating the multimodal risk knowledge graph with MLLM for risk decision-making generation involves several key steps. First, an improved DenseNet model is used to extract textual risk event entities from input risk images. Then, entity relationships are identified using a BERT+BiLSTM+CRF architecture in combination with a dictionary-driven relation extraction method. Next, the extracted image-based risk entities are linked to nodes in the knowledge graph. Relevant node information associated with the risk image is retrieved, along with additional entity-relation data extracted from the input risk query. Finally, the retrieved information is returned in the form of structured triples and used as prompt input to the MLLM for risk decision-making generation. The process of MLLM risk decision-making generation enhanced by the multimodal water conservancy project risk knowledge graph is illustrated in Fig 6.

### 4.2. Risk decision-making generation by fusing multimodal water conservancy project risk knowledge graph with MAAR

**Fig 7:**
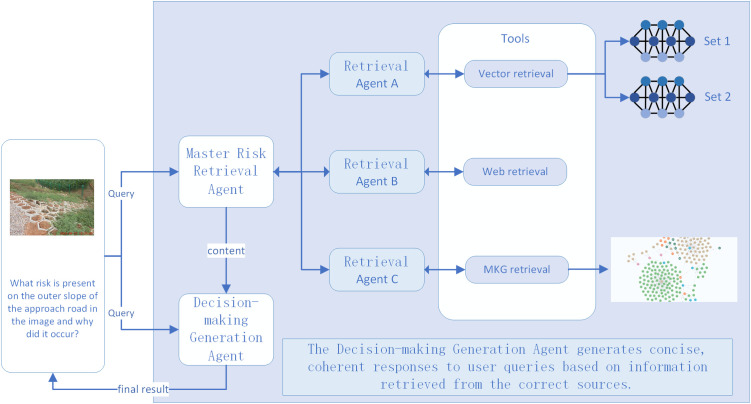
MAAR for water conservancy project risk decision generation scenario.

In complex water conservancy risk scenarios, relying on a single external knowledge source is insufficient to support comprehensive decision-making. From an engineering safety perspective, retrieval should not depend on one-time responses. The quality of retrieved contextual information must be validated or reasoned over to ensure the accuracy of final decisions. Agent represents the functional embodiment of LLM in specific roles and tasks, with the capability to access external tools and memory resources. Agentic RAG [29] integrates agents into the RAG pipeline, allowing coordinated execution across multiple components during generation. In water conservancy risk decision-making, the retrieval component interacts with various tools via agents to achieve autonomous information retrieval. To enable fine-grained risk perception and precise decision support, the multimodal water conservancy project risk knowledge graph is introduced as a core knowledge base. This approach mitigates the limitations of relying solely on text or structured data and enhances the comprehensiveness of risk identification. Fig 7 illustrates the whole process of MAAR for water conservancy project risk decision-making generation.

In Fig 7, the role of the risk decision-making generation agent is to produce the final response based on the user query. The master risk retrieval agent is responsible for assigning retrieval tasks according to the query context. This study does not introduce a retrieval discrimination agent to determine whether external knowledge is needed. Instead, it assumes that detailed risk information is always required by default. The detailed workflow of the multi-agent decision-making system is summarized in Table 3.

**Table 3:** Risk Information Query and Decision Generation Workflow.

Workflow: risk information retrieval and decision-making generation
**Water conservancy project risk query submission:** The water conservancy project risk query submitted by the user is received by the risk decision-making generation Agent and the master risk retrieval Agent. At this time, the master retrieval Agent acts as the core coordinator and assigns the risk query tasks to specialized retrieval agents based on the query requirements.
**Water conservancy project risk retrieval Agent:** The master risk retrieval Agent will assign the retrieval task based on the risk information and always prioritize the risk retrieval task to Agent C to query the multimodal water conservancy project risk knowledge graph. If the relevant risk information cannot be retrieved or the risk knowledge is insufficient, try to assign the retrieval task to Agent A. If the relevant risk information cannot be retrieved from the vector repository and the multimodal knowledge graph, try to assign the retrieval task to Agent B to use the Web retrieval tool.
** Agent A:** Semantic Retrieval, responsible for the retrieval of unstructured data from the Milvus Vector Library.
** Agent B:** Web Retrieval, focusing on searching for up-to-date risk decision-making information from the web or APIs.
** Agent C:** multimodal water conservancy project risk knowledge graph risk knowledge graph retrieval, responsible for retrieving structured information from the Neo4j graph database.
**Tool access and data retrieval:** During the water engineering risk retrieval process, each agent routes query requests to specific tools or data sources within its domain.
**Risk information retrieval results aggregation and validation:** The content retrieved by each sub-task is summarized to the master risk retrieval Agent, which verifies whether the retrieved results are relevant to the risk query entered by the user.
**Risk decision-making generation Agent generates response:** Based on the information from the master risk retrieval Agent, including similar semantically matched decisions, risk-related triples, fragmented information, etc., generates a concise and coherent response and feeds the response back to the user.

### 4.3. Risk decision-making system for water conservancy projects

**Fig 8:**
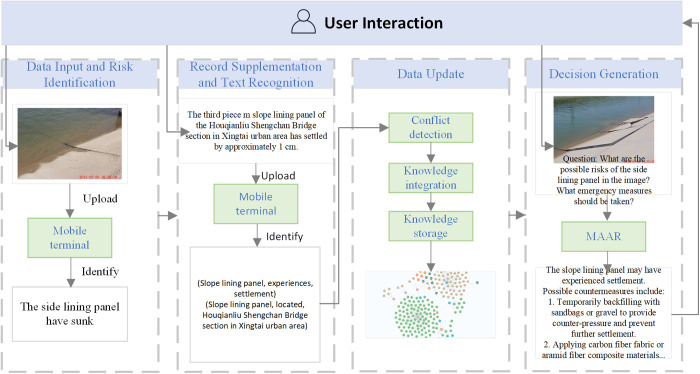
System application example diagram.

In the face of complex risks associated with water conservancy infrastructures, promptly identifying the type of risk that has occurred provides strong support for decision-making. By deploying the system on mobile terminals, it becomes possible to achieve accurate on-site risk identification. Field personnel can supplement inspection records based on the identified risks, including details such as the location, component, and specific characteristics of the risk. The realization of risk decision-making relies on the coordination of multiple procedural steps. The risk decision-making system for water conservancy projects encompasses the entire process from risk data input and update to final decision generation. The system architecture is illustrated in Fig 8, and the detailed implementation pathway is as follows:

Step 1: Data Input and Risk Identification

Risk images captured on site are uploaded to the mobile terminal, where an improved DenseNet-based risk image recognition model is first invoked for preliminary risk classification. The initially identified risks are processed in two ways: (1) After conflict detection, the results are directly linked to the corresponding risk images and integrated into the knowledge graph; (2) To obtain more comprehensive information, further details are supplemented to the initially identified risk entries.

Step 2: Record Supplementation and Text Recognition

The supplementary risk records include, but are not limited to, key information such as location, component, and specific details. These are manually added to complement content that cannot be automatically identified by the model. Once completed, the supplemented records are uploaded to the terminal. The BERT+BiLSTM+CRF-based entity recognition model and relation matching model are then used to extract risk entities and their corresponding relations from the text.

Step 3: Data Update

Based on whether detailed information has been supplemented or not, conflict detection is performed. The main types of conflicts include factual conflicts, logical conflicts, structural conflicts, and temporal conflicts. If no conflict is detected, the new entry is incorporated into the knowledge graph as the latest record. If a conflict is present, only the risk image is linked to the most similar existing node. During this process, knowledge fusion is also carried out using the aforementioned methods. All processed data are stored in a Neo4j graph database.

Step 4: Decision Generation

The MAAR-based decision generation method operates independently of any prerequisites and functions in parallel with other modules within the system framework. Even without data updates, the retrieval agents within MAAR continuously gather information from other data sources. When a user inputs a risk-related query into the decision-making module, MAAR returns the decision result accordingly.

### 5.1. Experiment preparation

The risk image data used in this study are sourced from the weekly inspection reports of the Middle Route Project of the South-to-North Water Diversion. 4,484 augmented water conservancy risk images are selected as the training dataset. These images cover 21 risks commonly encountered during project operation, including leakage, cracks, and settlement.

To enhance model robustness and reduce overfitting, the dataset is split into training and validation sets at a ratio of 8:2 prior to augmentation. Some textual data are paired with their corresponding risk images, forming image-text pairs used to support cross-modal alignment. To ensure consistency between the risk descriptions and the augmented images, the original image descriptions are rewritten using the AI Studio data annotation tool. Additional textual data are obtained from inspection reports and the Risk Prevention and Control Manual, which are refined by two hydrology experts. From this source, 595 risk decision-making records are selected as reference answers. Entity-relation pairs are extracted from these records and used to generate decision-making outputs for validation. Based on the prepared dataset, the following experiments are conducted:

1) Model comparison is performed against state-of-the-art image recognition architectures to evaluate the accuracy of the DenseNet-SHSA-CoordAtt model in risk image identification.
2) An ablation study is conducted to assess the effect of the alternating SHSA–CoordAtt fusion strategy by modifying how the modules are integrated into the DenseNet backbone.
3) A comparative analysis is carried out against traditional knowledge-enhanced approaches in water conservancy risk scenarios to evaluate the effectiveness of the multimodal knowledge graph in the MAAR framework for risk decision-making generation.

### 5.2. Experimental environment and model selection

All experiments in this study are conducted under the following environment: CPU (Intel® Xeon® Platinum 8352V), GPU (NVIDIA A800 80GB), Python version 3.11.11, CUDA version 12.6, PyTorch version 2.1.2, and VSCode 1.98.2 as the development editor. The baseline models used for risk image recognition include ResNet-50 [30], EfficientNet-V2 [31], RegNetY-400FM [32], and Vision Transformer-S [33]. For risk decision-making generation, the comparative methods include the original Qwen2.5-VL-7B, a RAG-based method built on Qwen2.5-VL-7B, and a knowledge-enhanced method integrating the multimodal water conservancy risk knowledge graph with Qwen2.5-VL-7B.

### 5.3. Experimental metrics

In this paper, for the risky image recognition model, the evaluation criteria are used as Accuracy, Precision, Recall and F1. The calculation formula is as follows:


$$Accuracy=\frac{TP\;+\;TN}{TP\;+\;TN\;+\;FP\;+\;FN}$$ (7)

$$Precision=\frac{TP}{TP\;+\;FP}$$ (8)

$$Recall=\frac{TP}{TP\;+\;FN}$$ (9)

$$F1=2\frac{Precision\cdot Recall}{Precision\;+\;Recall}$$ (10)

In the equation, TP is the number of samples that are correctly predicted to be positive in the actual positive category. TN is the number of samples that are correctly predicted to be negative in the actual negative category. FP is the number of samples that are incorrectly predicted to be positive in the actual negative category. FN is the number of samples that are incorrectly predicted to be negative in the actual positive category.

For MAAR the evaluation criteria used are BERT-score [34] and ROUGE-L [35]. BERT-score is based on the pre-trained Bert model, which is mainly used to measure the semantic similarity between the generated text and the reference text. ROUGE-L is based on the longest common subsequence (LCS) focusing on the content overlap and word order information. The calculation formula is as follows:


$$P_{BERT}=\frac{1}{\left|\hat{x}\right|}\sum_{{\hat{x}}_{j}\in \hat{x}}max_{x_{i}\in x}(x_{i}^{T}{\hat{x}}_{j})$$ (11)

$$R_{BERT}=\frac{1}{\left|x\right|}\sum_{x_{i}\in x}max_{{\hat{x}}_{j}\in \hat{x}}(x_{i}^{T}{\hat{x}}_{j})$$ (12)

$$F_{BERT}\;=\;2\frac{P_{BERT}R_{BERT}}{P_{BERT}\;+\;R_{BERT}}$$ (13)

In the equation, x is the text embedding given as reference and $\hat{x}$ is the text embedding generated by the model.


$$ROUGE-L[F_{lcs}]=\frac{(1\;+\;\beta^{2})R_{lcs}P_{lcs}}{R_{lcs}\;+\;\beta^{2}P_{lcs}}$$ (14)

In the equation, P is the ratio of the LCS length in the generated text to its own length, and R is the ratio of the LCS length in the generated text to the length of the reference text. β is an adjustable parameter used to adjust the relative weights of P and R.

### 5.4. Experimental results and analysis

#### 5.4.1. Validation of DenseNet-SHAH-CooradAtt based image recognition capability.

**Fig 9:**
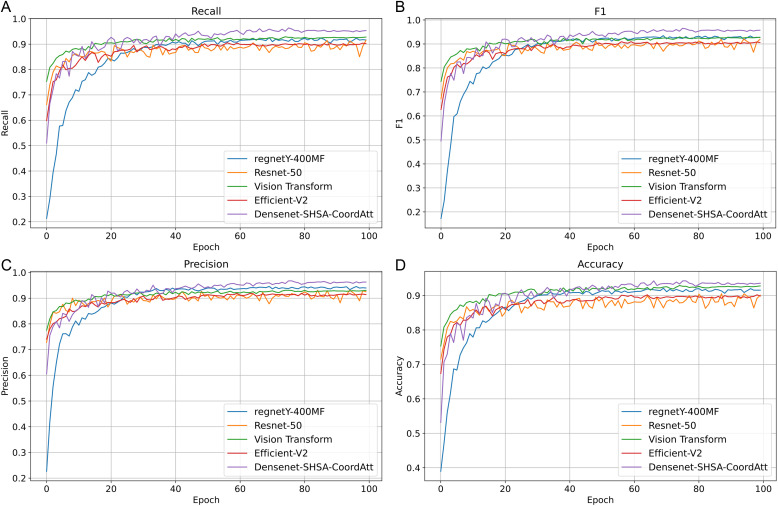
Changes in The Training Process of Each Assessment Metric for Different Model Validation Sets.

In order to validate the risk image recognition ability of DenseNet-SHSA-CoordAtt, this paper compares it with the average Recall, F1, Precision, and Accuracy of the baseline model on the risk image dataset. The specific comparison results are shown in Fig 9 and Table 4. As can be seen from Fig 9, with the growth of Epoch rounds, both the proposed model and the control model show a growth trend, but the convergence speed of the proposed model is significantly higher than that of the control model after 40 rounds of training. After the training reaches 100 rounds, the curve of the proposed model has been significantly higher than the control model. Through Table 4, it is found that although the Accuracy of ViT is slightly higher than that of the proposed model, the proposed model is much higher than ViT in terms of Recall, F1, and Precision indexes. For the water conservancy project risk image extraction capability, a single CNN or Self Attention based information extraction model may not be able to fully utilize the image’s complex features, thus limiting the accurate identification of risk information. Overall, the proposed model outperforms the above widely used models in risk image recognition, demonstrating the effectiveness of the proposed model for the image knowledge extraction task during the construction of the multimodal water conservancy project risk knowledge graph.

**Table 4:** Results of different model validation sets for each indicator.

Models	Average Accuracy	Average Precision	Average Recall	Average F1
Resnet-50	0.871	0.897	0.873	0.881
RegnetY-400MF	0.874	0.895	0.859	0.869
Efficientnet-V2	0.898	0.914	0.902	0.906
Vision Transformer	0.907	0.911	0.907	0.906
Densenet-SHSA-CoordAtt	**0.902**	**0.926**	**0.916**	**0.917**

#### 5.4.2. Impact of alternate fusion optimization strategies on the performance of the original model.

To evaluate the impact of the alternating fusion strategy of SHSA and CoordAtt on model performance, SHSA and CoordAtt modules are individually inserted at the beginning of each block within the original DenseNet architecture. The number of modules introduced in the single-module variants is kept consistent with that of the alternating fusion strategy. The resulting models are named DenseNet-SHSA and DenseNet-CoordAtt, respectively. As shown in Table 5, introducing only SHSA or only CoordAtt leads to limited performance gains compared to the alternating fusion strategy. Specifically, the recall and F1 scores improved by 1.3% and 1.0% over the baseline model. These results demonstrate that the alternating integration of SHSA and CoordAtt outperforms single-module enhancements.

**Table 5:** Results of module ablation on the validation set.

Models	Average Accuracy	Average Precision	Average Recall	Average F1
Densenet	0.895	0.922	0.903	0.907
Densenet-CoordAtt	0.898	0.923	0.909	0.911
Densenet-SHSA	0.900	0.923	0.909	0.911
Densenet-SHSA-CoordAtt	**0.902**	**0.926**	**0.916**	**0.917**

**Fig 10:**
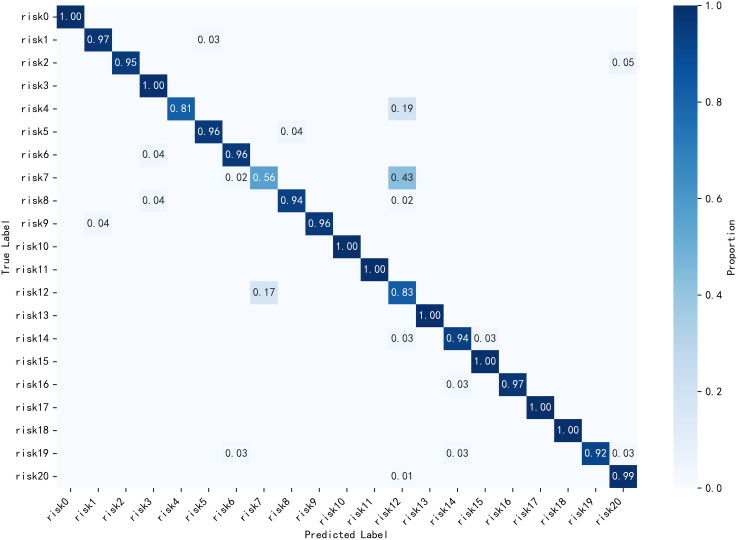
Densenet Confusion Matrix.

**Fig 11:**
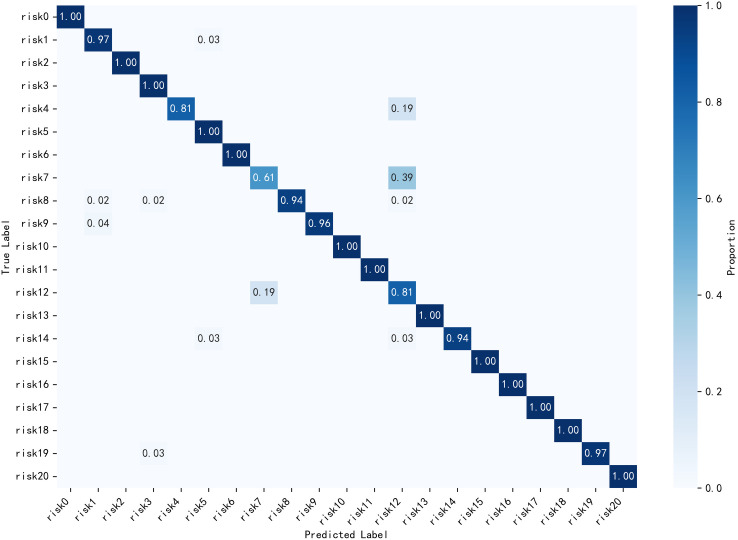
Densenet-CoordAtt-SHSA Confusion Matrix.

As shown in the confusion matrix in Fig 10 and Fig 11, the model exhibits poor discrimination between Risk 7 (dam isolation fence damage) and Risk 12 (dam isolation fence deformation), with a relatively high misclassification rate. Compared to the baseline model, the improved DenseNet-CoordAtt-SHSA reduces the error rate for this category by 4%. Additionally, the highest correct classification rate along the main diagonal improves by up to 5%.

#### 5.4.3. Effectiveness of multimodal water conservancy project risk knowledge graph in MAAR.

In order to test the effectiveness of multimodal water conservancy engineering risk knowledge graph in MAAR for risk decision-making generation, the 595 risk decisions screened in this paper are used as reference standards.Qwen2.5-VL-7B generates the results without referring to any professional information.Qwen2.5-VL-KG denotes the model enhanced by multimodal water conservancy project risk knowledge graph.Qwen2.5- VL-MAAR denotes the MAAR method without retrieving the multimodal knowledge graph.Qwen2.5-VL-MAAR-KG denotes the MAAR method that fuses the multimodal water conservancy project risk knowledge graph.

As can be seen in Table 6 the enhancement using external knowledge significantly improves the model’s expertise in answering within the domain when compared to the original generated model. Incorporating the multimodal water conservancy project knowledge graph into the MAAR, although the R-Bert metrics were slightly lower than the enhancement with the single use of the multimodal knowledge graph, P-Bert, F-Bert, and ROUGE-L were all improved. Compared to the MAAR without the retrieval of multimodal knowledge graph, the results of all the indicators are enhanced, which proves that the multimodal water conservancy project risk knowledge graph is effective for risk decision-making generation in the MAAR.

**Table 6:** Comparison of results of different enhancement strategies.

Models	P-Bert	R-Bert	F-Bert	ROUGE-L[F]
Qwen2.5-VL-7B	0.618	0.705	0.657	0.111
Qwen2.5-VL-KG	0.798	0.812	0.804	0.484
Qwen2.5-VL-MAAR	0.834	0.789	0.810	0.502
Qwen2.5-VL-MAAR-KG	**0.859**	**0.793**	**0.824**	**0.570**

## 5. Conclusion and outlook

Due to the variability of risk regions and the redundancy of background information in water conservancy imagery, traditional CNNs often struggle to maintain consistency between global and local semantic features while accurately extracting risk-related information. To address this issue, this study proposes an improved risk image recognition model, which significantly enhances the accuracy of risk region identification under complex visual conditions. Moreover, to overcome the limitations of traditional external vector databases used as third-party knowledge sources for LLMs—such as persistent hallucinations and flat information representation—this study constructs a multimodal water conservancy project risk knowledge graph and explores its integration within the MAAR framework for risk decision-making generation. Experimental results demonstrate that, compared with conventional single-graph enhancement methods, the proposed approach—combining the multimodal knowledge graph with a multi-agent agentic RAG strategy—achieves superior performance in risk decision-making generation. It notably improves both the accuracy and interpretability of generated content. This research provides a novel technical pathway for water conservancy risk management and highlights the potential of multimodal knowledge graphs in complex decision-making scenarios. Future work will incorporate additional modalities, including remote sensing and video data, to further enrich the construction of multimodal knowledge graphs for water conservancy applications. Furthermore, the scope of the research will expand beyond the South-to-North Water Diversion Project to cover a broader range of water conservancy infrastructures across the country, with the goal of supporting the sustainable, stable, and long-term development of national water projects.
